# Experimental investigations of crater formation as a result of high-velocity impacts on sand bed

**DOI:** 10.1371/journal.pone.0265546

**Published:** 2022-03-25

**Authors:** Rafał Mazur, Michał Beczek, Jacek Janiszewski, Wojciech Koperski, Cezary Polakowski, Bartosz Fikus, Agata Sochan, Ryszard Woźniak, Dawid Goździk, Magdalena Ryżak, Maciej Bańda, Andrzej Bieganowski

**Affiliations:** 1 Institute of Agrophysics, Polish Academy of Sciences, Lublin, Poland; 2 Faculty of Mechatronics, Armament and Aerospace, Military University of Technology, Warsaw, Poland; Soongsil University, REPUBLIC OF KOREA

## Abstract

The formation of craters is an important issue in the investigations of the surface of the earth and other planets. The aim of the study was to check whether the different textures of sand beds affect the size and dynamics of the formation of craters and ejection curtain after high-velocity impacts. The experiments were conducted using an aluminium impactor at two impact speeds (~700 and ~1300 m∙s^-1^) and a sand bed composed of either a broad range of sizes (<2.0 mm) or any of the three fractions obtained from it (<0.5, 0.5–1, 1–2 mm). The diameters, depths, wall slope, and rim heights of the resulting craters were measured. The ejecta curtain was characterized by the inclination angle of walls, base diameter, and expansion velocity. The mass of the transferred material and the depth of the impactor penetration were also determined. Additionally, the results were used to calculate dimensionless parameters commonly considered in crater studies (π_V_, π_2_ and α). The texture of the sand most clearly influenced the diameters of the craters, its effect could also be seen in the case of the distance covered by the ejected material. This information appears to be relevant for future research, providing some rationale to help assess in which aspects of the phenomenon the texture may be important.

## Introduction

The formation of the craters is an important issue in the investigations of the surfaces of the earth and other planets [1–3], in land engineering [4, 5] and in bulk materials engineering [6–8]. One of the mechanisms of crater formation is the impact of solid impactor [9, 10] or liquid drop [11, 12] on the granular bed. Different granular beds were investigated, for instance, model i.e. glass beads [13], sand [14], soil [15], gravel, or rocks [16]. Thus, this aspect is important in military research, with regard to the effectiveness of protective structures against bullets and shrapnel [17, 18].

The limiting factors for impact experiments are evidently the velocity of the impactor and the scale of the phenomenon. Therefore, investigations usually take into account the impact of small impactors in controlled laboratory conditions. The experiments described in the literature typically involve the use of different impactors released freely from a height or launched by different propulsion systems at various velocities [19–27]. Studies have been performed that focus on a range of different aspects including the formation of craters [22, 23], the effect of impact energy on penetration depth [19, 28, 29], the dynamics and behaviour of the impactor [30, 31], and the ejection of material during impact [25, 32, 33].

With respect to the velocity of the impactor, it is possible to divide existing work into those focussing on the impact of objects moving at low velocity (in the order of metres per second) and those examining objects moving at hundreds or even thousands of meters per second. In the former case, for example, Pacheco-Vazquez [27] investigated the ray systems and crater formations formed in a sand bed by different non-spherical projectiles (a clay ball with steel hemispherical protrusions) dropped from low heights (0.1–5.1 m). The author states that ray systems appear if the interface between the projectile contour and the granular surface has a shape that transfers nonradial horizontal momentum to the grains, which makes them converge as fine ejecta. Moreover, in the same study, it was observed that crater contours become circular independently of the projectile shape as the impact energy increases, which helps understand why most impact craters observed in nature have circular rims regardless of the shape of meteorites. Lohse et al. [34] described void collapse and jet formation processes following the impact of a steel ball dropped from heights of up to 1.5 m onto fine sand loosened by bubbling gas through it. Horabik et al. [7] performed experiments and discrete element method (DEM) simulations to study interactions in three-dimensional (3D) granular beds during low-velocity impact. In their experiments, a polypropylene ball was released freely from heights of 0.11–0.9 m onto a glass bead pack.

In the case of high-velocity (>100 m∙s^-1^) and hypervelocity (>1500 m∙s^-1^) impacts, measurements invoke high stresses and high strain rates [29]. As such, the behaviour of target materials under extreme loading can cause particle crushing under high stress, shear localisation, and pore fluid pressure accumulation. Stöffler et al. [20] describe cylindrical Lexan projectiles striking a non-cohesive quartz sand bed, using a vertical ballistic gun to achieve impact velocities of 5900–6900 m∙s^-1^. They quantitatively measured the distribution and properties of the ejected material, and the dimensions of craters produced. Using an electro-discharge launcher, Savvateev et al. [19] achieved projectile velocities of 4000 m∙s^-1^, focussing on the depth of impactor penetration into a sand bed. These authors state that the depth of penetration is impeded by the melting of the projectile material. Therefore, they concluded that, for investigations at high velocities, the use of refractory materials (such as tungsten alloy) is the best way of achieving greater depths. They also proposed the most suitable projectile shape, namely a rod with the aspect ratio λ = 5–7 (i.e. the ratio between the projectile length and diameter), as larger aspect ratios can result in bulking, bending, and breaking of the impactor. Bless et al. [35] observed the sand penetration of projectiles with different noses (ogive, hemispherical, and conical) fired at velocities between 600 and 2200 m∙s^-1^. Along with penetration depth, these authors also studied the defragmentation of projectiles and demonstrated that, above 770 m∙s^-1^, impactors begin to break up and penetration decreases drastically. Jakielaszek et al. [36] experimentally and numerically investigated the interaction between penetrators and sand beds with modified geometrical and mass characteristics using the Smoothed Particle Hydrodynamics method (SPH). They confirmed the applicability of their methods of estimating the target characteristics (structure and properties) to ensure required resistant force vs. time patterns at a relatively low-impact-velocity of circa 200 m·s^-1^. The correlation between hypervelocity and crater size (depth and diameter) has also been investigated. Since the early 1960s, the crater penetration depth has been described using the power law of impact velocity [37–39]. A different approach was proposed by Baker and Persechino [40], who suggested that crater diameter is described by a linear function of impact velocity. This was further expanded on by Baker [41], who compared the linear and power function models of crater diameter for microparticle impacts with velocities ranging from 2500 to 27000 m∙s^-1^. Baker stated that the linear model was more suitable but the crater shape factor should be taken into account as this varies with impact velocity. It is worth noting that the development of models and formulas aimed at increasingly accurate descriptions of impact phenomena remains a focus of much present-day research [42, 43].

A review of the literature has highlighted gaps in understanding the influence of specific sand bed properties, such as grain size, on crater formation and ejected material during high-velocity impact. This is important, since such knowledge could form the basis for: i) planning experiments (i.e. the results may provide some rationale to help decide in which aspects of the phenomenon the texture of the granular bed may be considered as one of investigated factors), ii) interpretation of experimental results and models (including those used to validate existing models and develop future models), and iii) interpretation of real existing craters. To address this, the present study aimed to characterise bed surface deformation processes during high-velocity impacts and describe the formations created in sand beds with different size fractions.

## Methodology and methods

### Sand beds

Alluvial quartz sand collected from the central section of the Vistula River Valley (51°24’56"N, 21°56’33"E) near Góra Puławska, Poland, was used in the experiments. The authors declare that no specific permissions were required for this sampling location and confirm that the field studies did not involve endangered or protected species. The particle size distribution of the base material based on sieve analysis after removal of the skeleton particles (>2.0 mm) was as follows: 62.0% fraction <0.5 mm, 34.5% fraction 0.5–1.0 mm and 3.5% fraction 1.0–2.0 mm. The tested material was sieved to obtain the following three fractions: <0.5 mm; 0.5–1 mm; 1–2 mm. Unfractionated sand was also tested, which was sieved through a 2 mm mesh sieve to remove plant residues, other impurities, and a small amount of gravel (hereafter referred to as the <2 mm fraction). Gradation curves of the fractions used are presented in S1 Fig. The bulk density (measured with Powder Tester PT-S, Hosokawa Micron B.V., Netherlands), internal friction angle (measured with ring shear tester RST-01.pc, Schulze Schuettgutmesstechnik, Germany) were determined for each fraction (Table 1). The coefficients of curvature and uniformity were calculated based on the BS EN 933–1 standard [44]. Also the shape of the grains based on circularity and elongation parameters [45] was determined using a Morphologi G3 microscope (Malvern, UK) at 50 × magnification.

**Table 1:** Physical properties of the tested bed material.

Fraction [mm]	Internal friction angle [°]	Particle size distribution [mm]			Coefficient of		Fraction shape parameters		Bulk density of the formed bed [g∙cm^-3^]
		D(10)	D(50)	D(90)	Curvature	Uniformity	Circularity	Elongation	
<0.5	32.67 ± 0.32*	0.26	0.34	0.43	1.00	1.64	0.89	0.23	1.65 ± 0.02*
0.5–1.0	34.97±0.3*	0.59	0.66	0.79	0.95	1.34	0.91	0.20	1.74 ± 0.001*
1.0–2.0	36.37±1.47*	1.16	1.30	1.49	0.98	1.28	0.89	0.21	1.67 ± 0.01*
<2.0	34.4±0.7*	0.26	0.40	0.58	0.96	1.95	0.89	0.23	1.75 ± 0.01*

All measurements made to obtain the data presented in this table were carried out on independent small amounts of the tested sand fractions prepared exactly in the same way as used for the impacts.

* ± standard deviation of 3 replications.


$$Circularity:\theta =\frac{4\pi A}{P^{2}}$$ (1)

$$Elongation:\varepsilon =1-\frac{W}{L}$$ (2)

where *A* is the area of the grain [mm^2^]; *P* is the perimeter of the grain [mm]; *L* is the length of the grain [mm]; and *W* is the width of the grain [mm].

The sand beds were loosely packed in plastic containers (the sand was poured into the container in small portions slowly and from a low height, which was similar to the height of sand pouring in the bulk density measurement apparatus) with sloping walls with the following dimensions: height = 285 mm; upper outer diameter (at the surface) = 620 mm; bottom diameter (at the base) = 390 mm. The size of the containers was chosen so as not to restrict the emerging craters. After being arranged centrally under the propulsion system, the levelling of the container was controlled (the area on which the bed was placed was levelled before measurements) and excess material was gently removed from the surface using a long smooth wooden strip.

### High-velocity propulsion system

**Fig 1:**
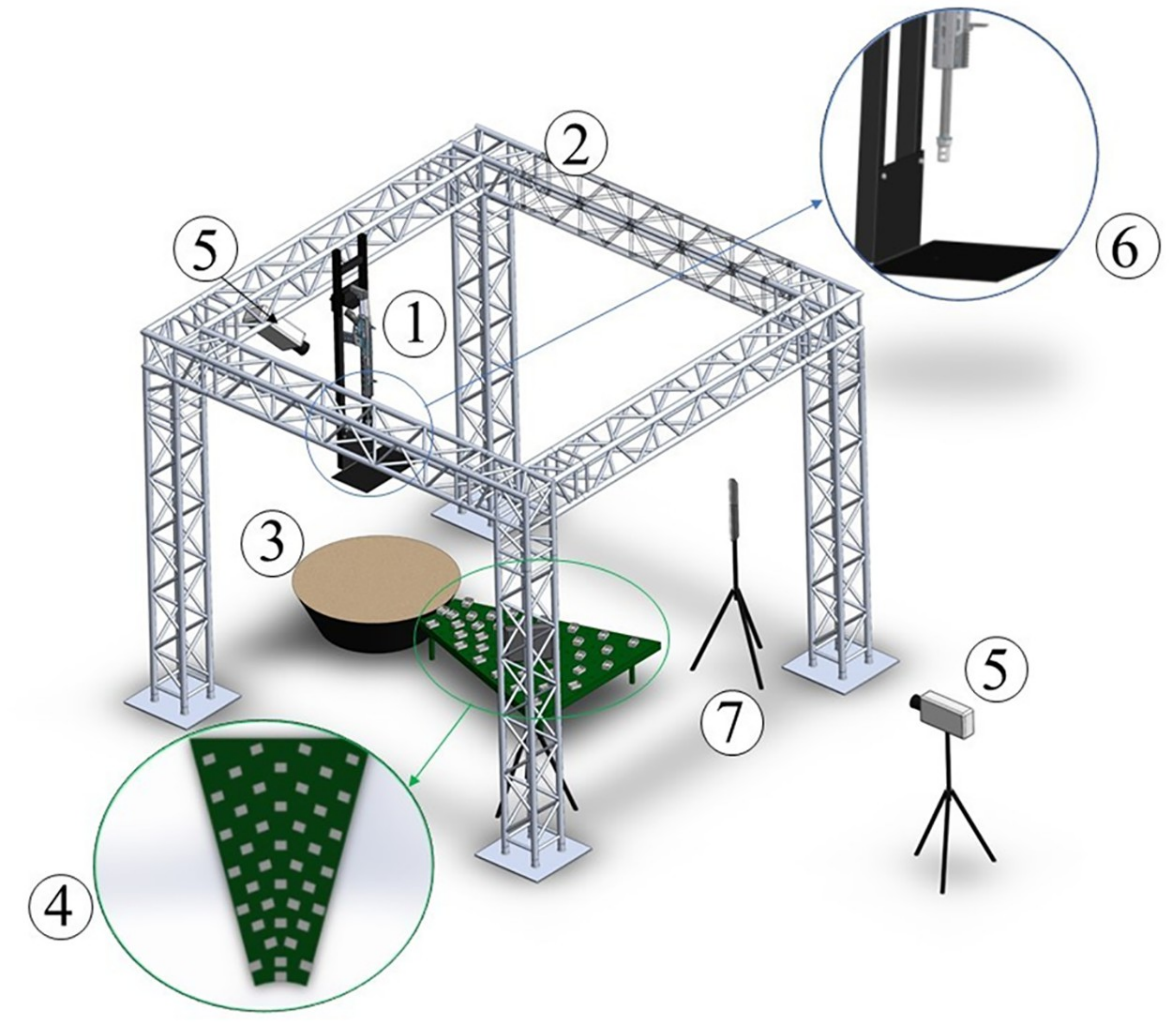
Measurement stand set-up. 1–rifle; 2–structure to ensure rifle stability during the shot; 3–sand container; 4–table with containers for collecting ejected grains of sand; 5–high-speed cameras; 6–protection plate against propellant gases; 7–front lighting LED lamp.

The measurement stand for high-velocity impacts was built at the Military University of Technology in Warsaw (Fig 1). The propulsion system was suspended on a structure that ensured adequate stability. The barrel was pointed vertically down, aimed at the central part of the sand bed, and the muzzle (barrel outlet) was positioned 1.5 m above the bed surface. A steel plate with an orifice mounted below the muzzle protected the sand surface from the wave of expanding propellant gases. The plate was located 1.3 m above the bed surface. This value was adjusted experimentally in order to reduce the blast wave effects. Additional drawings of the measurement stand set-up (detailed views and sections) are presented in S2 Fig.

The impactors were made of aluminium alloy, cylindrical with a base diameter of 8 mm and a length of 16 mm, and weighed 2.17 g. The velocity of the impactors was adjusted by the mass of the propellant charge in the round case. The experiments carried out assuming two velocities (hereafter referred to as 1300 m∙s^-1^ and 700 m∙s^-1^). Due to technical limitations (even minimal differences in powder alignment or compaction could affect the acceleration of the impactor), we were not able to ensure repeatable absolute speeds. Therefore, the actual speeds were 1321 ± 94 m∙s^-1^ and 690 ± 61 m∙s^-1^. These average speeds (and calculated standard deviations) were determined on the basis of measurements made with the use of high-speed cameras for each individual shot. For each variant of the experiment (i.e. for each sand fraction and impactor velocities), the measurements were carried out in five repetitions.

### Impact deformation measurement

**Fig 2:**
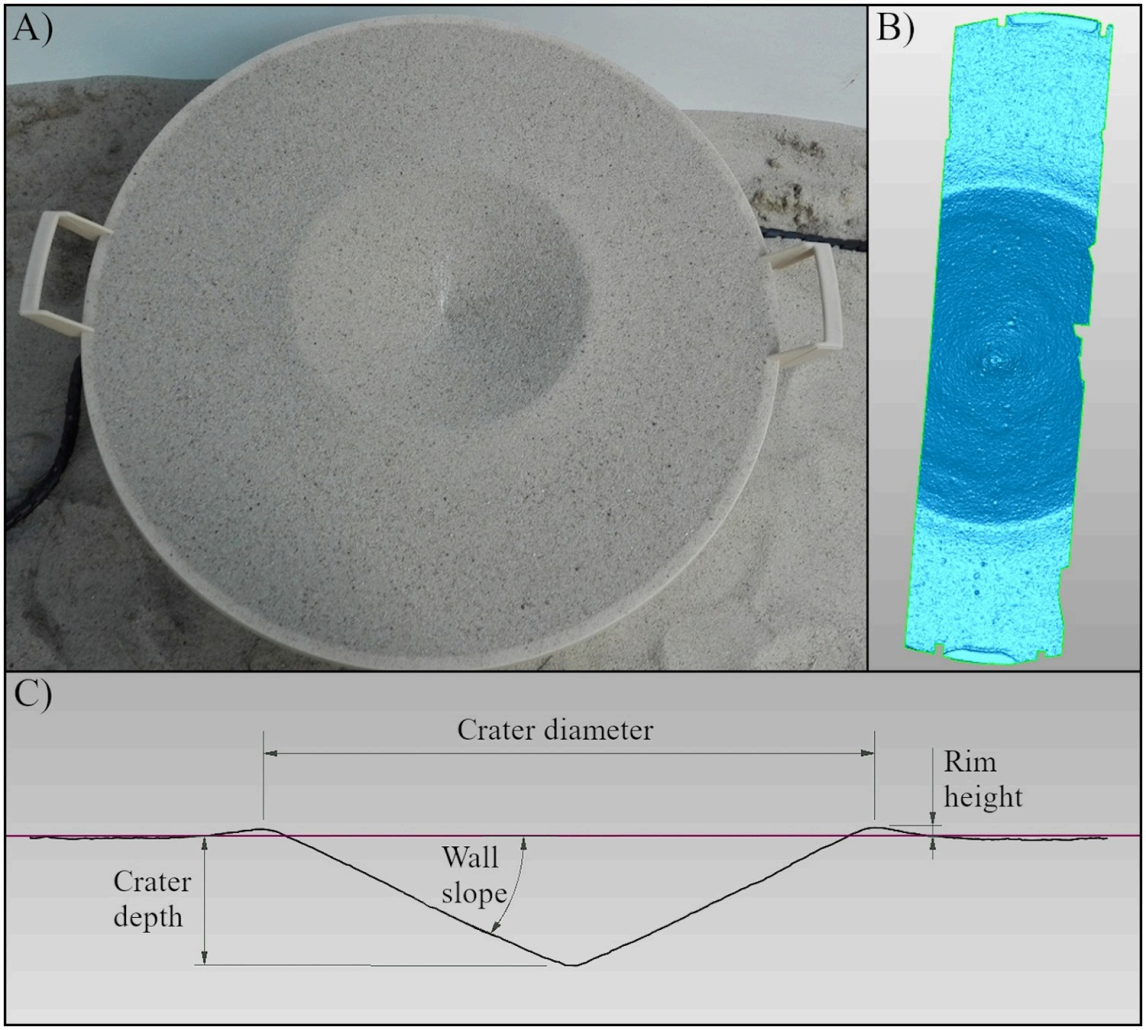
Elements used in the crater analysis. (A) example crater after the impact at a velocity of ~700 m∙s^-1^ into a <2 mm fraction sand bed; (B) example of a crater section analysis; (C) cross-section of a crater with bed deformation parameters indicated.

A surface scanner (Scan3D UNIVERSE 10 MPiX, Smarttech 3D, Poland) was used to measure bed deformation following impact. An example of an impact crater is shown in Fig 2A. The scanner resolution was 0.039 mm (655 pt·mm^-2^) and the device was calibrated before the measurements were made. Each scan generated a point cloud with a measurement space of 150×100×90 mm. Considering the size of the craters, mapping the bed surface required scans to be merged, which after appropriate arrangement, covered an area circa 150 mm wide passing through the centre of the crater and ending at the edges of the bed container. Further measurements of the scanned section (Fig 2B) were also performed to characterise the regular shapes of the deformations. These measurements and the point cloud processing were carried out using Smarttech3D measure software (Smarttech, Poland). Crater dimensions were analysed using Geomagic Control 2015 (3D Systems, USA), which allowed the determination of (a) rim height; (b) crater depth (measured relative to the level of sand bed before impact); (c) crater diameter; and (d) crater wall slope (Fig 2C).

### Impact imaging with high-speed cameras

The impacts were recorded using two monochromatic high-speed cameras. The first camera, a Phantom V1612 (Vision Research, USA), was positioned 7 m from the centre of the bed, perpendicular to the direction of the impactor flight. This camera recorded at 20000 frames per second (fps) with a resolution of 768×768 pixels and shutter time equal to 15 μs. It was used to determine the velocities of the projectiles and for the description of the ejecta curtain. Front lighting was provided by two LED lamps (7000 lumens). The second additional camera, a Phantom Miro V310 (Vision Research, USA), was placed 3 m above the bed (Fig 1). It recorded at 3200 fps with a resolution of 1280×800 pixels and shutter time equal to 60 μs. This camera allowed checking the centricity of the impacts (only central impacts were taken into account) and facilitated observations of the sand-slumping process (also called sand avalanches) that occurred from the walls during the final stage of the crater formation. The recordings and analyses were performed using Phantom Camera Control (PCC) software. An example of a video from the high-speed camera measurements is presented in S1 Video.

**Fig 3:**
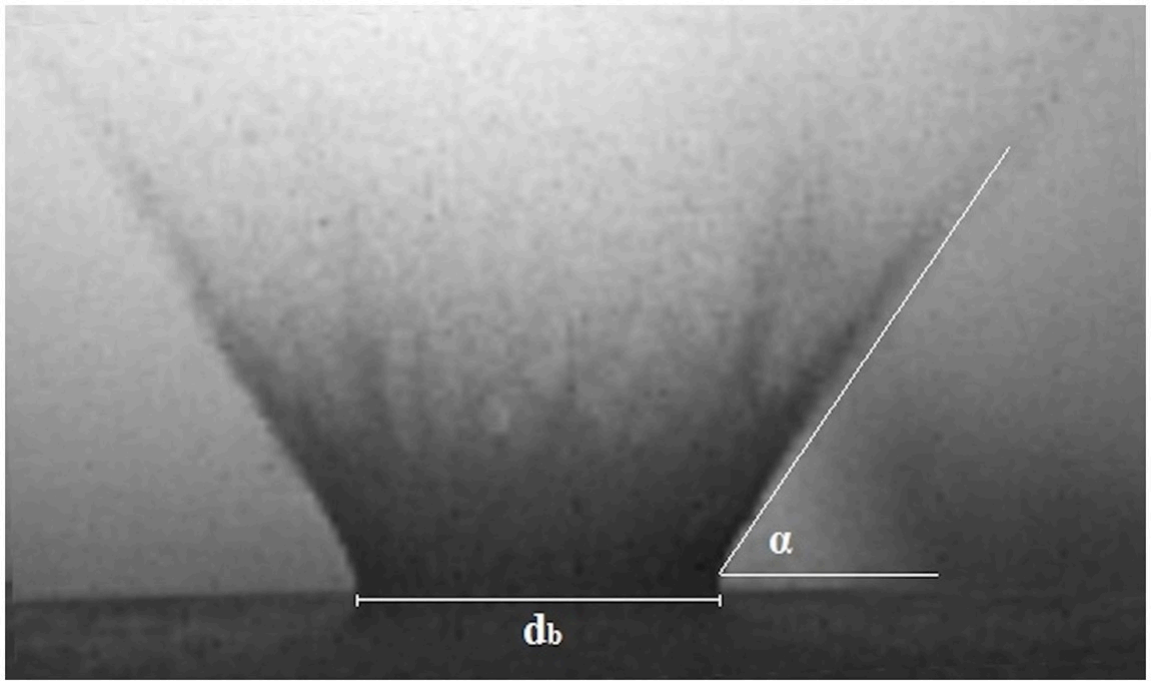
Description of the ejecta curtain based on high-speed camera imaging. Symbols: d_b_−base diameter, α –angle of curtain wall.

The images recorded by the first camera were calibrated from px to mm dimensions, and used for the parameterization of the curtain formed during the impact. In order to examine the dynamics of this aspect, the following moments in time were chosen (image frames counted from the moment of reaching the sand surface by the impactor): 0.005, 0.01, 0.015, 0.025, 0.05, 0.075, and 0.1 s. Based on the applied methodology, the specified quantities were measured:

1. the angle of inclination of curtain walls (the average value of left and right walls in all analysed moments in time) (Fig 3),
2. the base diameter of the curtain (Fig 3),
3. the curtain expansion velocity (the velocity at which the base of the curtain moves outwards in the radial direction). The velocity was estimated by fitting a power law in time to the base diameter in a least-square sense (*f* = *a∙t*^n^) and defined as the time derivative of the curtain radius value as the function of time.

### Ejecta mass and impactor penetration depth measurement

A set of plastic containers (8 × 7 cm) used as ‘splash boxes’ was positioned starting from the edge of the bed container (Fig 1). The boxes were set at intervals of 7 cm (counting from the center of each container). The top edge of the containers was at the same level as the bed surface. This set-up allowed the collection of sand grains ejected beyond the edge of the bed container during impact, i.e. distances greater than 310 mm from the impact site. The sand collected in the containers was weighed (±1 mg) after each shot. Taking into account the mass of sand accumulated in the cups and the part of the surface covered at particular distances, the amount of sand thrown over individual distances was determined on the basis of proportions. The sum of the results for all distances gave an estimate of the total mass of ejected sand. Based on the location of the last cups in which a measurable amount of sand (>1mg) was found, the maximum distance of sand transport was determined.

For each test, the impactor penetration depth was determined by removing the sand covering the impactor using a vacuum with a small stream of air, so that the projectile position was not altered. The impactor depth was measured from the upper edge of the sand container to the upper edge of the impactor residue using a calliper.

### Statistics

Data were analysed using Statistica 13.1 software. The Shapiro–Wilk test was used for checking the normality of data. Statistically significant differences were determined based on Analysis of Variance (ANOVA) and Tukey tests with a significance level (p) of 0.05.

## Results

### Characteristics of craters

**Fig 4:**
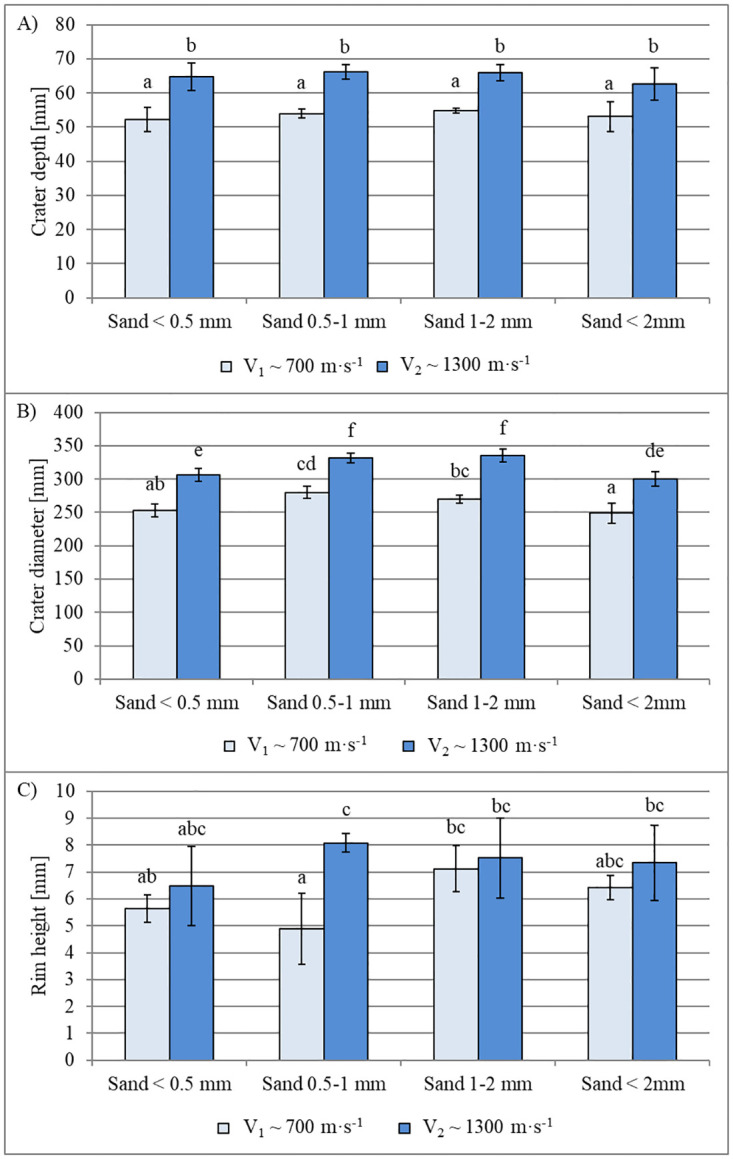
Crater descriptors. (A) maximum crater depth; (B) crater diameter; and (C) maximum rim height. Data are mean values ± standard deviation. The letters refer to the statistical comparison (post hoc Tukey’s HSD test): the same letters–no statistically significant differences.

The characteristics of the craters formed by the impacts of the impactors on the sand beds with different grain-size distributions and at different velocities are shown in Fig 4.

The depths and crater diameters varied significantly depending on impactor velocity; the higher the impactor velocity, the greater the crater depth and diameter. The same relationship was observed for rim height, although this was only statistically significant for the 0.5–1 mm fraction.

The data presented in Fig 4A indicate that for the tested impactor velocities, crater depth did not depend on the size of the sand grains. For a projectile moving at a velocity of ~1300 m∙s^-1^, the crater depths ranged from 62.6 mm ± 4.8 mm (for the <2 mm fraction) to 66.2 mm ± 2.1 mm (for the 0.5–1 mm fraction). For a projectile velocity of ~700 m∙s^-1^, the crater depths were noticeably shallower and ranged from 52.2 mm ± 3.5 mm (for the <0.5 mm fraction) to 54.8 mm ± 0.8 mm (for the 1–2 mm fraction).

A different trend was observed for the crater diameter (Fig 4B); the craters for the 0.5–1 mm and 1–2 mm fractions were statistically significantly wider than for the <0.5 mm fraction and unfractionated (<2 mm) sand (for lower velocity, the difference between fractions 1–2 mm and <0.5 mm vanishes). At velocities of ~1300 m∙s^-1^, crater diameters ranged from 300.2 mm ± 10.6 mm (for the <2 mm fraction) to 335.4 mm ± 9.9 mm (for the 1–2 mm fraction). At the lower projectile velocity (~700 m∙s^-1^), crater diameter ranged from 248.8 ± 15.0 mm (<2 mm fraction) to 280.0 mm ± 9.1 (0.5–1 mm fraction).

According to the obtained results, it does not appear that the bed grain size or projectile velocities (in the range considered in our experiment) influence the height of the crater rim. The average rim height ranged from 4.9 mm ± 1.3 mm to 8.1 mm ± 0.3 mm. The slope angles of the crater walls ranged from 23.4 ± 0.9° to 27.2 ± 2.2° but not differ statistically between the bed gain size and impactor velocity experiments.

**Fig 5:**
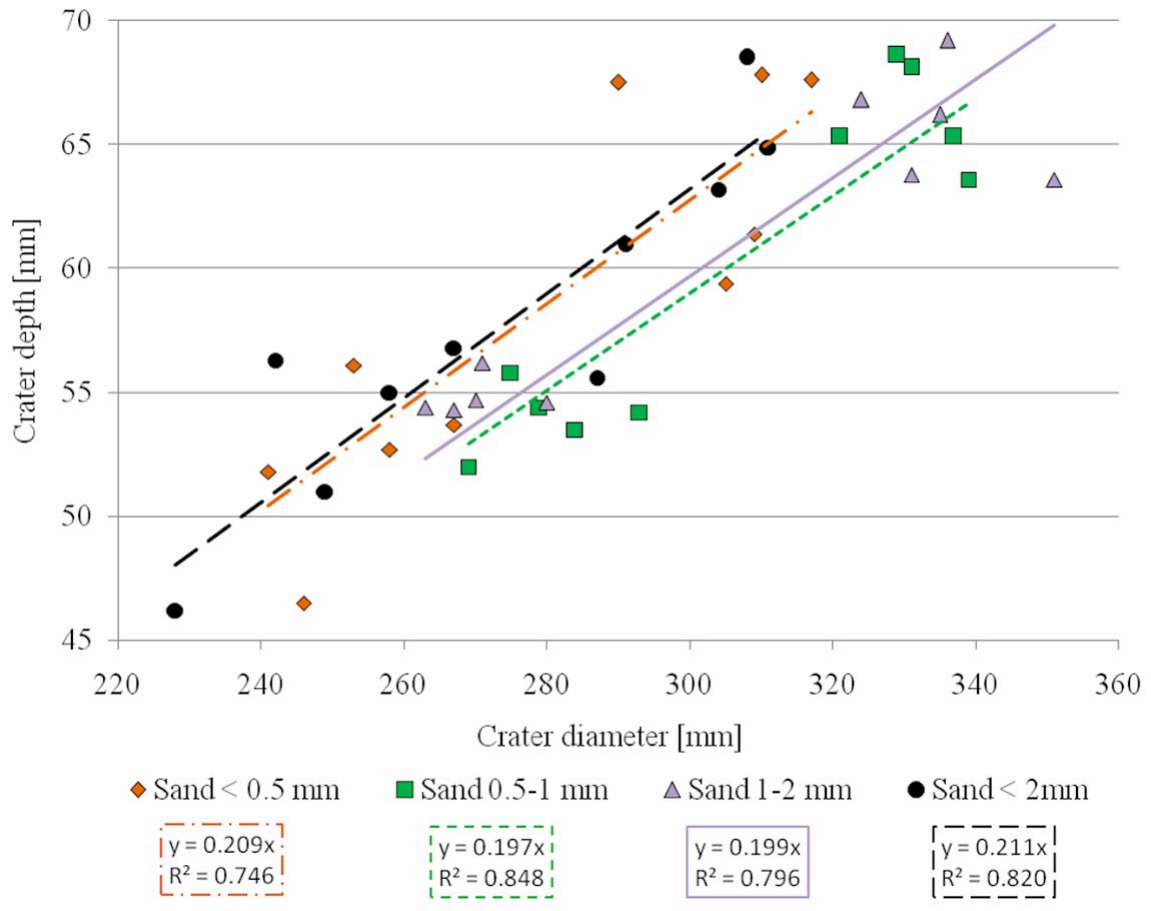
Depth-to-diameter ratio of craters for individual sand fractions. By interpolating straight lines, the intersection at the origin of the graph was forced. R^2^ represents the coefficient of determination.

One of the most common parameters used in literature is the crater depth-to-diameter ratio, which has been used to describe craters of different origin and with different degradation properties [46–49]. The correlation between crater depth-to-diameter ratio, impactor strike velocity, and sand bed grain size is shown in Fig 5. The results are grouped in two areas that reflect impactor velocity; however, the straight lines approximated for each grain-size fraction in Fig 5, irrespective of impactor velocity, are almost parallel. In other words, the depth-to-diameter ratio (c 0.2) of the craters seemed to be constant over the investigated range of impactor velocities for the particular sands. Additionally, it should be noted that the values of the slope of the crater and the crater depth-to-diameter ratio are related. The inclusion of both qualities was motivated by the intention to facilitate the interpretation of the presented results (without the need to recalculate them).

### Characteristics of ejecta curtain

**Fig 6:**
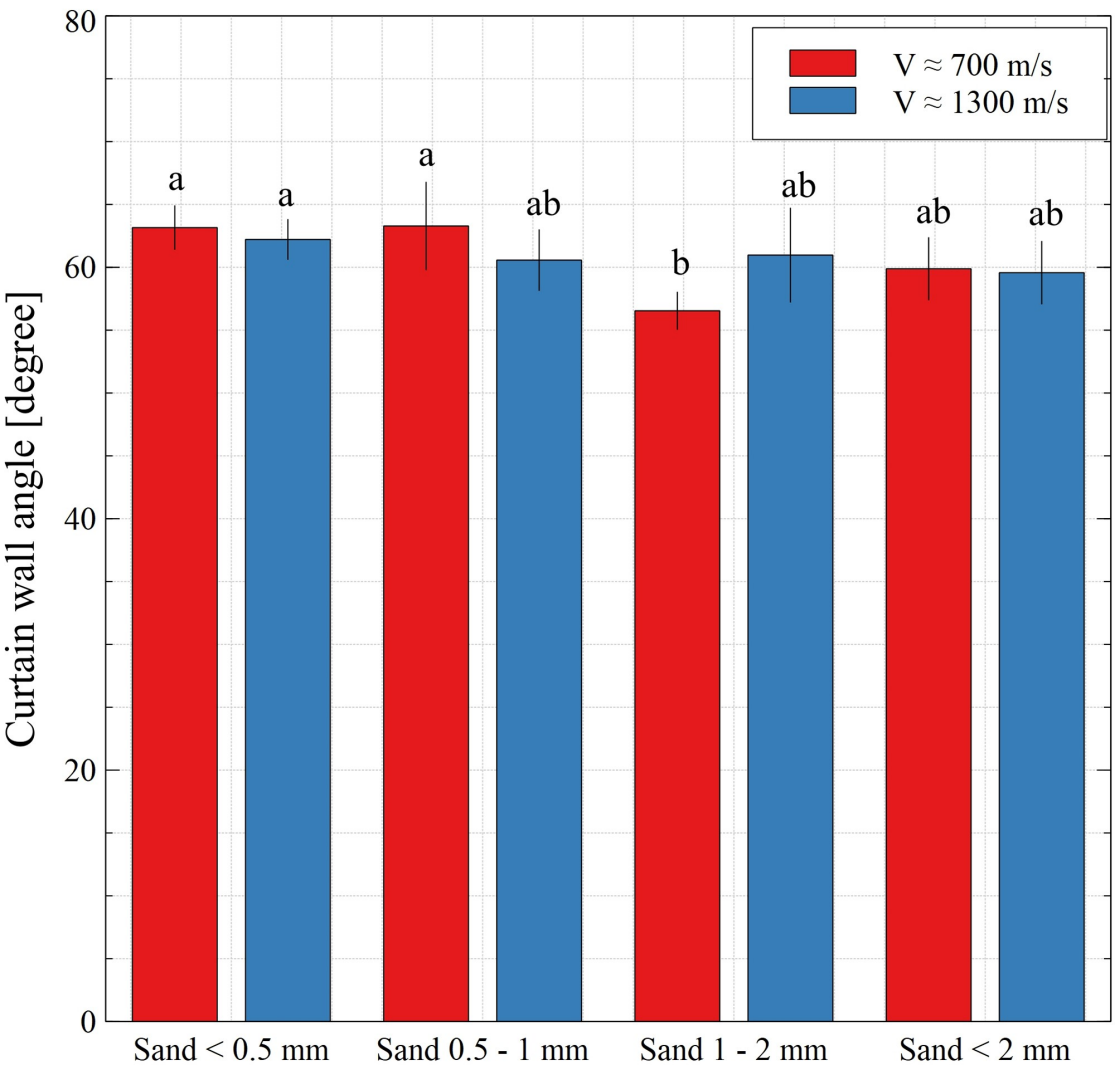
Average angle of inclination of the curtain walls. Data are mean values ± standard deviation. The letters refer to the statistical comparison (post hoc Tukey’s HSD test): the same letters–no statistically significant differences.

**Fig 7:**
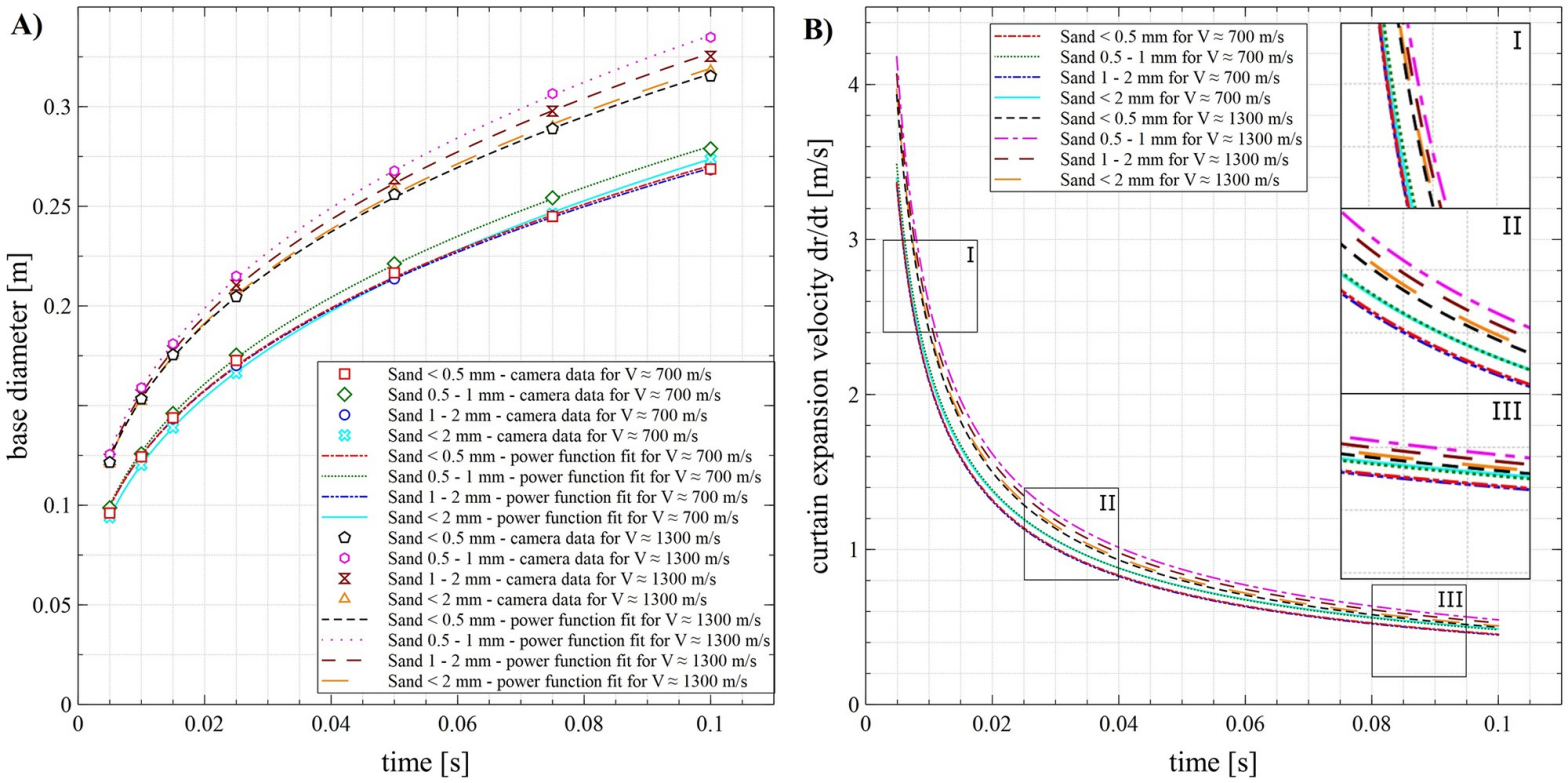
Curtain descriptors. (A) base diameter of the spreading curtain in subsequent moments in time; (B) curtain expansion velocity in subsequent moments in time. Data are mean values ± standard deviation. The standard deviation bars on graphs are very small and coincide with the symbols. There are no significant differences between the sand fractions in each moment in time step.

The measured quantities of the ejecta curtain formed by the impacts on the sand beds with different grain-size distributions and at different velocities are shown in Figs 6 and 7.

Based on the data presented in Fig 6, it can be said generally that the angle of formation of ejecta curtain walls did not depend on the projectile velocity and size of the sand grains (in almost all investigated cases, there were no statistically significant differences). The values of the angle for both velocities ranged from 56° to 63°.

The influence of the impactor velocity was observed in the base diameter of the curtain (Fig 7A). The measured values in each moment in time were significantly different, comparing each fraction in both velocities. However, in both cases, the effect of the sand fraction was insubstantial: in each moment in time, the values for the different fractions were generally not significantly different. In the measured moments in time (from 0.005 s) for a projectile moving at a velocity of ~1300 m∙s^-1^, the base diameter ranged from 120 mm to 334 mm and for the projectile velocity of ~700 m∙s^-1^, the values were lower and ranged from 93 mm to 273 mm.

In the early stage of the phenomenon (near the moment in time of 0.005 s), the curtain expansion velocity (Fig 7B) was dependent on the impactor velocity. Here, the highest values recorded: for a projectile moving at a velocity of ~1300 m∙s^-1^, the expansion velocity at 0.005 s was in the range of 3.9–4.2 m∙s^-1^ and for a projectile velocity of ~700 m∙s^-1^ it was about 3.3–3.5 m∙s^-1^. In the later stages, the differences between the impactor velocities were mitigated, and the curtain expansion velocity was very similar for both variants. Despite the impactor velocity, there were no statistically significant differences between the sand fractions in each moment in time.

### Characteristics of ejecta mass and distance

**Fig 8:**
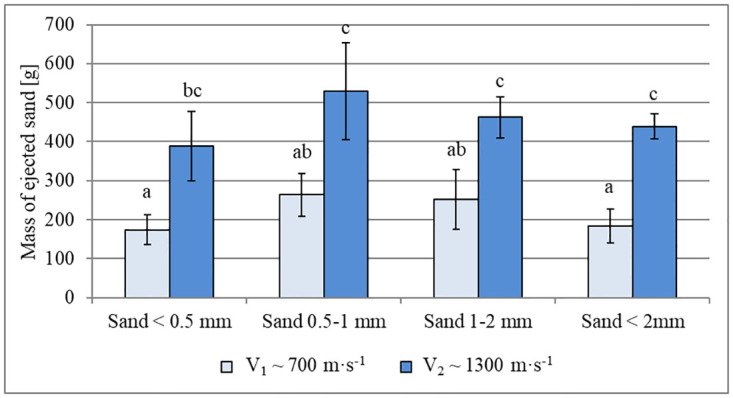
Estimated mass of ejecta transported beyond the bed container (i.e. >310 mm from the impactor strike) for individual tested bed grain-size fractions. Data are mean values ± standard deviation. The letters refer to the statistical comparison (post hoc Tukey’s HSD test): the same letters–no statistically significant differences.

The mass of sand grains ejected beyond the bed container is shown in Fig 8, which varied significantly depending on impactor velocity. With an impact velocity of ~1300 m∙s^-1^, the highest recorded mass of ejecta was 530 g ± 124 g for grains with a diameter of 0.5–1 mm. At a velocity of ~700 m∙s^-1^, for all fractions the mass of the ejected material was approximately half of that. No significantly differences in ejecta mass were observed between the different sand fractions at higher or lower impact velocities (Fig 8).

The maximum distances over which sand grains were moved are listed in Table 2. Depending on the distance moved, material was captured by multiple containers (more for longer distances), although the containers in the outer row covered only 0.02% of the total ring area (Fig 1). These data are therefore considered to be more qualitative rather than quantitative, although some clear relationships can still be identified.

**Table 2:** Maximum distances from the impact point to which sand was moved during impactor strike.

Impactor velocity [m·s^-1^]	Fraction [mm]	Flight range [cm]
~1300	<0.5	157–164
	0.5–1	164–171
	1–2	199–206
	<2	164–171
~700	<0.5	94–101
	0.5–1	129–136
	1–2	157–164
	<2	108–115

As the ejected grains were captured in 7cm wide splash boxes, the maximum distance is expressed in 7 cm intervals.

The grains of sand ejected outside of the beds travelled shorter distances when impacted with less energy, i.e. in the lower impactor velocity experiments. With respect to grain size, the coarser the fraction, the further ejected grains travelled. This is best seen when the three fractions are considered together, as the unfractionated sand (<2 mm) makes interpretation more difficult given the mix of small and large grains it contained. In the case of the unfractionated bed, the ejected grains over longer distances were invariably the larger ones.

### Characteristics of impactor depth

**Fig 9:**
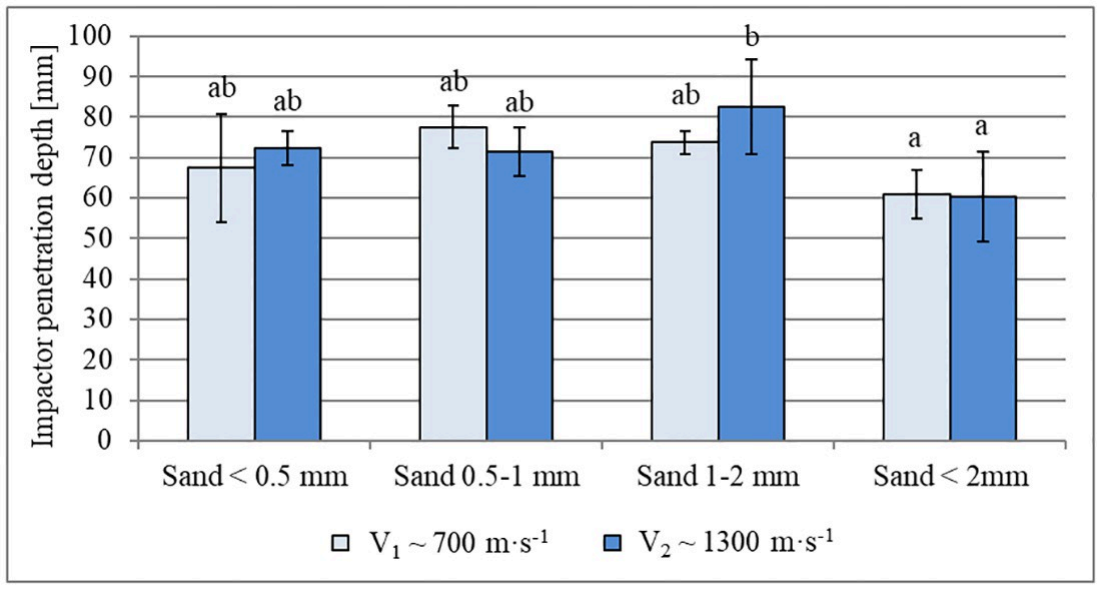
Impactor penetration depth in sand beds with varying grain sizes. Data are mean values ± standard deviation. The letters refer to the statistical comparison (post hoc Tukey’s HSD test): the same letters–no statistically significant differences.

The depths of the impactors after striking the beds of different grain sizes and at the two velocities are shown in Fig 9. The average penetration depth at an impact velocity of ~1300 m∙s^-1^ ranged from 60.3 mm ± 11.2 mm (<2 mm fraction) to 82.5 mm ± 11.9 mm (1–2 mm fraction). At an impact velocity of ~ 700 m∙s^-1^, penetration depth ranged from 60.9 mm ± 6.1 mm (<2 mm fraction) to 77.5 mm ± 5.1 mm (0.5–1 mm fraction). The differences in penetration depth between impactor velocities were not significant, however. In other words, at the velocities applied, the penetration depth was not dependent on the energy of the impactor. Furthermore, for both velocity variants, the only statistically significant differences between the fractions occurred comparing the unfractionated sand (<2 mm) with the fractions for which the highest impactor penetration depth value was found (1–2 mm fraction for higher velocity). Noteworthy is the presence of grains of sand that have been fragmented by the impact in the impactors’ direct vicinity and the penetration of the impactor.

## Discussion

In this study, the bed material was sampled from one place and then fractioned with sieves. All of the sand was obtained from the same source, grain shapes (based on circularity and elongation measurements, Table 1) were similar between the different beds irrespective of the size fraction. As the impact tests were performed on air-dry material, pore-water did not influence the results. Instead, differences in the structure of the beds would have affected their bulk density, which might explain the results obtained. When a relatively large space is created between large grains, these gaps can be filled with small grains when these are present. In such cases, there are more sand grains per unit volume and, consequently, bulk density is higher. This is shown in Table 1 where the bulk density was the highest for the unfractioned sand, which contained both larger- and smaller-sized grains. In contrast, the coarser fraction (1–2 mm) had the lowest bulk density.

### Crater

Bed deformation was assessed using the four parameters of crater diameter, depth, wall slope, and rim height. The depths and diameters of the craters after impactor strike varied significantly with impactor velocity (Fig 4A and 4B). This is expected given the significant difference in kinetic energy in each case (1,893 J at an impact velocity of ~1300 m∙s^-1^ compared to 517 J at a velocity of ~700 m∙s^-1^). The effect of the impactor velocity on crater dimensions has already been addressed in investigations conducted by Schmidt [50] as well as Schultz and Gault [51], whose results are consistent with the relationships presented in our study.

No differences in crater depth were observed in the beds with grain sizes under each impact velocity condition (Fig 4A). This was most likely due to the slumping of sand into the transient crater, which is an unstable cavity, momentarily formed by the ejection of material during impact. The high-speed recordings made from above the impact bed showed that for each tested fraction, grains of sand slumped from the inside walls of the crater and covered the impactor. This process altered the initial crater depth and the diameter and slope of the crater walls. Changes in the shapes of craters during their formation have been experimentally and numerically studied [22, 52], and the results of these works are consistent with our observations. Based on the video material, it can be assumed that the depth of the transient crater was equal to the depth at which the impactor stopped. A similar assumption has been provided by Yamamoto et al. [53] for craters formed in a granular deposit (glass beads). After sand rearrangement stopped, the crater took its final and stable form, which was the point at which the 3D scan was obtained.

Based on Fig 4B, in the case of impacts in beds with larger sand grains (i.e. 0.5–1 mm and 1–2 mm), the crater diameters were statistically significantly larger at each impact velocity. This can partly be explained by the mass of ejected material (Fig 8). Despite the lack of statistically significant differences, a broad trend was observed for the displacement of higher amounts of material from the beds with larger grains. Importantly, our experiments did not measure material movement within the bed container where, according to Wünnemann et al. [25], the largest amount of ejected material is deposited; however, based on the observed trend, the susceptibility of grains to being ejected as a function of size had at least a partial influence on the extent of bed deformation. It also cannot be ruled out that the observed variation in crater diameter was affected by the friction between grains, variations in the primary crater depth, and the sand-slumping process, which may have been slightly different in the individual beds. Ultimately, it is not possible to definitively determine the reasons for the diameter variations, likely being a result of a combination of bed properties. A similar argumentation based on the influence of several factors on the crater diameter was presented by Yamamoto et al. [53]. The experiment performed by these authors included the impacts on targets consisting of glass spheres with different sizes. Their results were consistent with ours, where the larger diameters of craters were associated with the bigger sizes of elements forming the deposit. Housen and Holsapple [16] reported that even a significant change in the ratio between the diameter of the impactor and the diameter of deposit grains (in their experiment this parameter ranged from 0.32 to 50) had a minor influence on the crater profile. In the case of experiments that were the subject of this study, where the difference in crater diameters for the different fractions forming the deposit was noticeable, this ratio ranged between 4 and 16.

A relationship similar to the crater diameter was observed for rim height whereby higher velocity impacts resulted in higher rims; however, rim height differences between the two impact velocities were only significant for the 0.5–1 mm fraction. This might be explained by the wide data-scatter shown by the error bars in Fig 4C. Indeed, the deviation of the measurements may have resulted from the small heights of the rims (6–8 mm) in relation to the size of sand grains (for the coarser fraction up to 2 mm). With this ratio, given the high-resolution measurements of the scanner, even single grains located at the top of the rims could significantly affect the obtained values.

The slope of the crater walls did not differ between size fractions or impactor velocities. Taking into account the similarities between the grains forming individual fractions and the sand slumping from the crater walls, similar angle values seem unsurprising. Although Miura et al. [54] demonstrated that angle of repose values depend on the fraction size (lower angles were obtained for smaller fractions), they also found that angle depends on the mass of falling sand. Similar angle values were presented by Al-Hashemi and Al-Amoudi [55], although they suggest that the angle of repose decreases with an increase in particle size.

When comparing crater dimensions between studies, it is important to note that the phenomena discussed have been studied under specific conditions including impactor shape and velocity, physical target properties, and ambient pressure conditions. Craters similar in size to those we observed are reported by Wünnemann et al. [25], with diameters ranging from 31.85 to 33.65 cm, depths ranging from 5.3 to 7.4 cm, and a maximum rim height of 0.9 cm. These results were obtained using an aluminium projectile with a mass of 0.38 g accelerated to 6000–8000 m∙s^-1^, based on experiments performed in the 1970s including those of Stöffler et al. [20]. The diameters of craters reported by Schmidt and Housen [21] using an aluminium projectile with a mass of 0.315 g and an impact velocity of circa 5000 m∙s^-1^ averaged 20 cm. A similar diameter was obtained when a twice as heavy projectile was used at a lower velocity of circa 2 km∙s^-1^. Cintala et al. [56] observed craters with diameters of 13.9–18.2 cm using an aluminium ball with a diameter of 4.76 mm and a sand bed with a 1–3 mm grain diameter and impact velocities of 802–1920 m∙s^-1^. Tsujido et al. [33] reported crater diameters of circa 50 mm following the impact of a 3 mm aluminium impactor travelling at circa 200 m∙s^-1^.

### Dimensionless scaling of the crater

To compare our findings with the results obtained by other authors, the dimensionless scaled crater volume (also known as cratering efficiency) and scaled gravity (also known as the inverse Froude number) should be used. These non-dimensional parameters can be expressed by the following formulas [57]:

$$\pi_{V}=\frac{\rho_{sand}V_{crater}}{m_{proj}}$$ (3)

$$\pi_{2}=\frac{gr_{proj}}{v_{proj}^{2}}$$ (4)

Where ρ_sand_ is the bulk density of the fractions [kg∙m^-3^]; V_crater_ is the crater volume [m^3^]; m_proj_ is the impactor mass [kg]; g is the Earth’s gravity [m∙s^-2^]; r_proj_ is the impactor radius [m]; and v_proj_ is the impactor velocity [m∙s^-1^].

**Fig 10:**
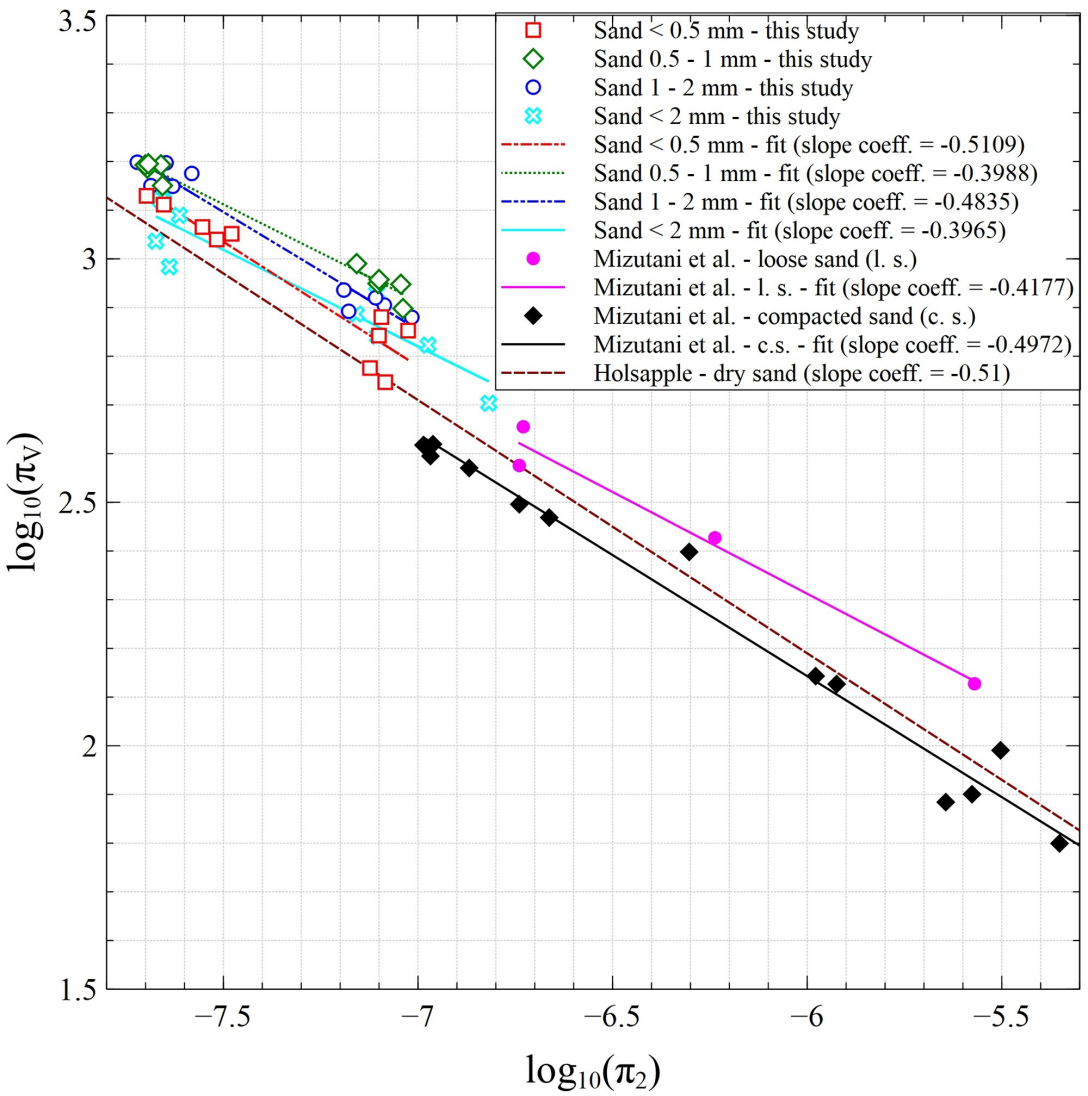
Dependence of the dimensionless scaled crater volume on non-dimensional scaled gravity.

The comparison of the crater volumes obtained in our research with results presented by Mizutani et al. [58] and Holsapple [57] is presented in Fig 10. In the presented analyses, the values of the data required for particular repetitions (i.e. individual impactor velocity and crater volume) were taken into account to calculate π_V_ and π_2_.

The dashed line in Fig 10 is taken from the work of Holsapple [57]. It presents the relationship for sand; however, there was no information about the investigated sand fractions. The lack of this information causes a problem with a direct comparison with our results. Nevertheless, it can be seen that the slope coefficient is the most similar for the relationship of our fraction <0.5 mm.

A better comparison of our result is possible with the data presented by Mizutani et al. [58], as the authors provided much more information about the measured sand beds. The experiments were carried out by these authors using sand with an average size of 0.2 mm and aluminium impactors varying in size, considering e.g. cylinders with a diameter and length of 10 mm. As presented in Fig 10, the non-dimensional crater volumes estimated in our study are comparable with results reported by the authors and exhibit a similar power-law exponent (slope in the presented double logarithmic plot). This applies in particular to sand <0.5 mm, which was the closest to the loose sand used by Mizutani et al. [58]. It can be seen that our results for sand <0.5 mm and the results for loose sand obtained by Mizutani et al. [58] form a straight line. In other words, our findings complement the results shown by Mizutani et al. [58] at the smaller impactor velocities.

The analysis of the lines in Fig 10 revealed that, except the line for loose sand from Mizutani et al. [58], both other lines from literature, i.e. for compacted sand [58] and for dry sand [57], our results are shifted up. This shift could be related to compaction of the sand bed in the experiments presented by the other authors. As demonstrated by Mizutani et al. [58], the compaction of the sand results in a different crater shape (not conical in this case). If so–the volume of the crater may be different from that calculated with the assumption of the cone shape, which may influence the π_v_ value.

Based on our results, crater depth-to-diameter ratio (often referred to as the crater aspect ratio α) remained almost constant (at 0.2) irrespective of impactor velocity and sand grain size. This is well illustrated in Fig 5 where two distinct groups of points can be seen that represent craters with smaller diameters and shallower depths formed by the ~700 m∙s^-1^ impacts and those with larger diameters and deeper depths formed by the ~1300 m∙s^-1^ impacts. These findings are consistent with the α value obtained for simple craters reported by Collins et al. [52]. Similar results are also reported by Zhao et al. [47], who tested the impact of a drop of water on a bed of glass beads with a diameter of 90 μm to imitate sand. Irrespective of kinetic energy (i.e. the falling height of the water drop up to 12 m), these authors obtained a linear relationship between crater depth and diameter, where α = 0.20 ± 0.01. These results are consistent with our observations, despite notably different measurement conditions (e.g. drop impact vs. solid body impact, and large differences in impact velocity). Furthermore, Daubar et al. [46] and Agarwal et al. [49] report similar α values for craters on the Moon, Mars, and Mercury, although their diameters and depths are several orders of magnitude larger. Moreover, similar ratios have been noted by Schmidt and Housen [21] and Cintala et al. [56], who obtained an α value of circa 0.23 for a 1–3 mm sand bed and 0.17 for 0.25 mm fine sand. The value of the coefficient obtained in our study was also comparable to the coefficient determined for loose sand beds in Mizutani et al. [58] experiments, which was 0.25.

### Ejecta curtain

The ejecta curtain was characterized by three quantities: angle of curtain walls, base diameter, and curtain expansion velocity. Before the discussion, it has to be mentioned that the above quantities were analysed in different moments in time starting from 0.005 s. Since the ejecta curtain was poorly visible in the initial stage of formation (up to 0.005 s), these analyses were omitted. The reason for this was the difficulty in defining a consistent edge of the curtain resulting from the sand grains ejected at different angles at this stage and the curved part of the curtain base called the ‘neck’ [33].

The angle of forming ejecta curtain walls (Fig 6A) did not depend on the projectile velocity and the size of sand grains, as indicated by the statistical analysis. This is not surprising, as the values of this parameter are related with the slope (angle) of the crater walls. As reported by Tsujido et al. [33], the angle of the curtain depends on the ejection angle of sand grains. In the analysed moments in time (from 0.005 s), the grains that compose the curtain are ejected from the inside of the transient crater at an angle that depends on the slope of the walls. Since we were not able to measure the slope of the crater during the subsequent formation stages, we can only suppose that they may have been similar to the angle of the curtain. The values of the curtain angle (56°–63°) are consistent with results presented by Deboeuf et al. [59], who found in their experiment that the angle was approximately constant in time and equal to ∼56°. This was also confirmed by Tsujido et al. [33], where the authors obtained values of the curtain angle ranging from 52° to 63°, depending on the projectile density.

In relation to Fig 6B, the measured base diameter of the curtain in the different moments in time was only dependent on the projectile velocity. Due to the considerably higher amount of energy supplied to the sand bed by the projectile at a higher velocity, the base diameter was bigger in the first analysed stage of curtain formation (comparing both velocities). Thus, it seems that the difference in the diameters is mostly influenced by the initial stage of the phenomenon after the impact–the higher impact energy resulted in the more dynamic spreading of curtain walls in the 0.005 s (Fig 6C). However, in the subsequent stages of expansion, the velocity of the curtain decreased substantially and was similar for both variants of impactor velocity. This seems to have been related to the internal friction of the sand grains and air resistance. It is worth noting that the base diameter of the curtain in the analysed moments in time may be related to the diameter of the transient crater during the formation process.

### Ejecta mass and distance

It is not surprising that the mass of the ejected material depended on impactor velocity. According to Fig 8, almost doubling the velocity of the projectile (from ~700 m∙s^-1^ to ~1300 m∙s^-1^) resulted in an almost two-fold increase in the mass of the ejected sand for each of the fractions. It can, therefore, be concluded that the amount of material displaced largely depended on the amount of energy applied to the bed by the projectile.

Statistical analysis did not reveal significant differences in ejecta mass between the individual sand fractions. However, based on the average values, for both velocity conditions, more material was ejected from the beds composed of large grains (0.5–1 mm and 1–2 mm). Furthermore, comparing the volume of the craters formed with the mass of ejected sand, for each experimental condition, the vast majority of the displaced material remained within the bed container, which is consistent with the observations of Wünnemann et al. [25].

Noteworthy, for both impactor velocities, the relationship between the maximum ejection distance and the grain size showed that the coarser fractions were moved over greater distances (Table 2). This can be explained primarily by the deceleration caused by drag (air resistance) during the flight of sand grains. This deceleration is approximately proportional to the ratio of the grain cross-sectional area to its volume [60]. Assuming a spherical grain shape, based on simple geometrical relationships, this dependency is inversely proportional to the grain radius. So it is expected that deceleration of the smallest fraction will be circa four times that of the coarser fraction. Moreover, we anticipate that, depending on the grain size, the material ejection process (grain acceleration) will vary due to the different damping properties of the momentum transfer inside the sand bed. For example, damping in the smaller grain-size fraction bed would have been greater due to the larger number of contacting particles and larger contacting surface area. In analytical models of terminal ballistics, this effect is mainly taken into account in the form of a linear velocity-dependent term defining resistance during the projectile penetration.

### Impactor depth

Under both velocity conditions, the impactor penetration depth was only statistically different between the unfractionated sand (<2 mm) and the fraction for which the greatest penetration depth was found (1–2 mm fraction for higher velocity). The shallower penetration into the unfractionated sand may reflect its higher bulk density as previously noted; greater grain packing likely results in greater bed resistance, impeding the penetration of the impactor.

The lack of statistically significant differences between impactor penetration depths under the two velocity conditions is somewhat surprising. This may be partly explained by the strength of the impactor material, i.e. aluminium. According to Savvateev et al. [19], above a certain critical velocity, bed penetration is limited by the specific material used to build the impactor as the thermal softening and melting process is dependent on the material melting point. Using steel projectiles (with a melting point of circa 1500°C), these authors found that the depth of the bed penetration was limited above a velocity of circa1600 m∙s^-1^ in the case of a ball and circa 2000 m∙s^-1^ in the case of a very thin and short cylinder. Savvateev et al. [19] also reported observations for the impact of a 4.8 g aluminium projectile at a velocity of 1950 m∙s^-1^ with the same length-to-diameter ratio (2:1) as in the projectiles used by us. Under these conditions, a penetration equal to circa 50 mm was observed, which is similar to the results of our measurements. Compared to our study, the impactors used by Savvateev et al. [19] had a 130% higher mass, 56% greater cross-sectional area, and 50% higher impact velocity. For such projectiles a greater penetration depth would typically be expected. However, the reported penetration depths exceeded the critical melting velocity threshold, particularly given the melting point of aluminium is circa 660°C and the volumetric heat capacity of aluminium is circa 60% that of steel. Furthermore, Savvateev et al. [19] suggest that in the case of projectiles with smaller mass, the critical velocity may be lower, which would also be consistent with our results.

Another phenomenon that should be included in this part of the discussion is the projectile rebound. The rebound can occur during crater formation and involves a reversal of the projectile’s direction and its ejection above the crater bottom. Guzman et al. [61] report that rebound can affect the difference between the maximum and final projectile penetration and has already occurred at projectile velocities of 150 m∙s^-1^. Furthermore, if a rebound occurs, the initial velocity of the projectile will affect the temporary maximum penetration of the deposit, but its importance for the final position of the projectile will be significantly reduced. As suggested by Kondic et al. [62], increasing the initial velocity of the projectile may increase the rebound time. The occurrence of this phenomenon during our experiments cannot be excluded, especially that the depth of the layer under which we found our projectiles was approximately equal to their diameter, which, according to the information presented by Guzman et al. [63], corresponds to the coverage of the impactors for which a rebound was observed.

Penetration depth can also be affected by “false nose” or “kernel” formations. According to Omidvar et al. [29], the false nose is formed in the sand in front of a projectile and takes the shape of a cone. These formations are thought to be created by fairly small projectiles (<30 mm) impacting at high velocity, which is consistent with our experimental conditions.

Unfortunately, we are not able to assess to what extent the above-mentioned factors and/or their combinations had a significant influence on the depth of penetration and the course of the investigated phenomenon.

## Conclusions

We investigated the influence of sand bed textures and impactor velocities on the size and dynamics of surface deformation (craters) and ejection curtain. The most pronounced effect of the grain size occurred in the case of the crater diameter. In contrast to the above observations, the crater depth, the crater diameter, and the mass of ejected sand varied significantly with the impactor velocity, but this did not affect the penetration depth or the crater rim height. Irrespective of the impactor velocity and the grain-size properties of the sand bed, the crater depth-to-diameter ratio (aspect ratio, α) remained relatively constant at 0.2. The values of the dimensionless parameters (π_V_ and π_2_) indicate compliance of the results of our measurements with the trends observed in other studies. Although a minor number of significant differences in the crater and curtain shape were found, it should be noted that, the coarser fractions were transported over the greatest distances in all cases. The results presented in our work ensure better understanding of projectile impacts on granular beds and can be used in modelling of these phenomena.

## Supporting information

S1 Fig Gradation curves of sand fractions. The dashed lines mean different sand fractions.
(TIF)

S2 Fig Detailed drawings of the measurement stand set-up: Cross sections (a, b) and plan view (c). 1–rifle; 2–structure to ensure rifle stability during the shot; 3–sand container; 4–table with containers for collecting ejected grains of sand; 5–high-speed cameras; 6–protection plate against propellant gases; 7–front lighting LED lamp.
(TIF)

S1 Video Example of a video of the high-velocity impact on the sand bed recorded with the use of high-speed cameras. (MP4)
