# Supplementary figures and images for: Experimental investigations of crater formation as a result of high-velocity impacts on sand bed
Source: PLoS One. 2022 Mar 25;17(3):e0265546. doi: 10.1371/journal.pone.0265546 (PMC8956193; doi:10.1371/journal.pone.0265546)

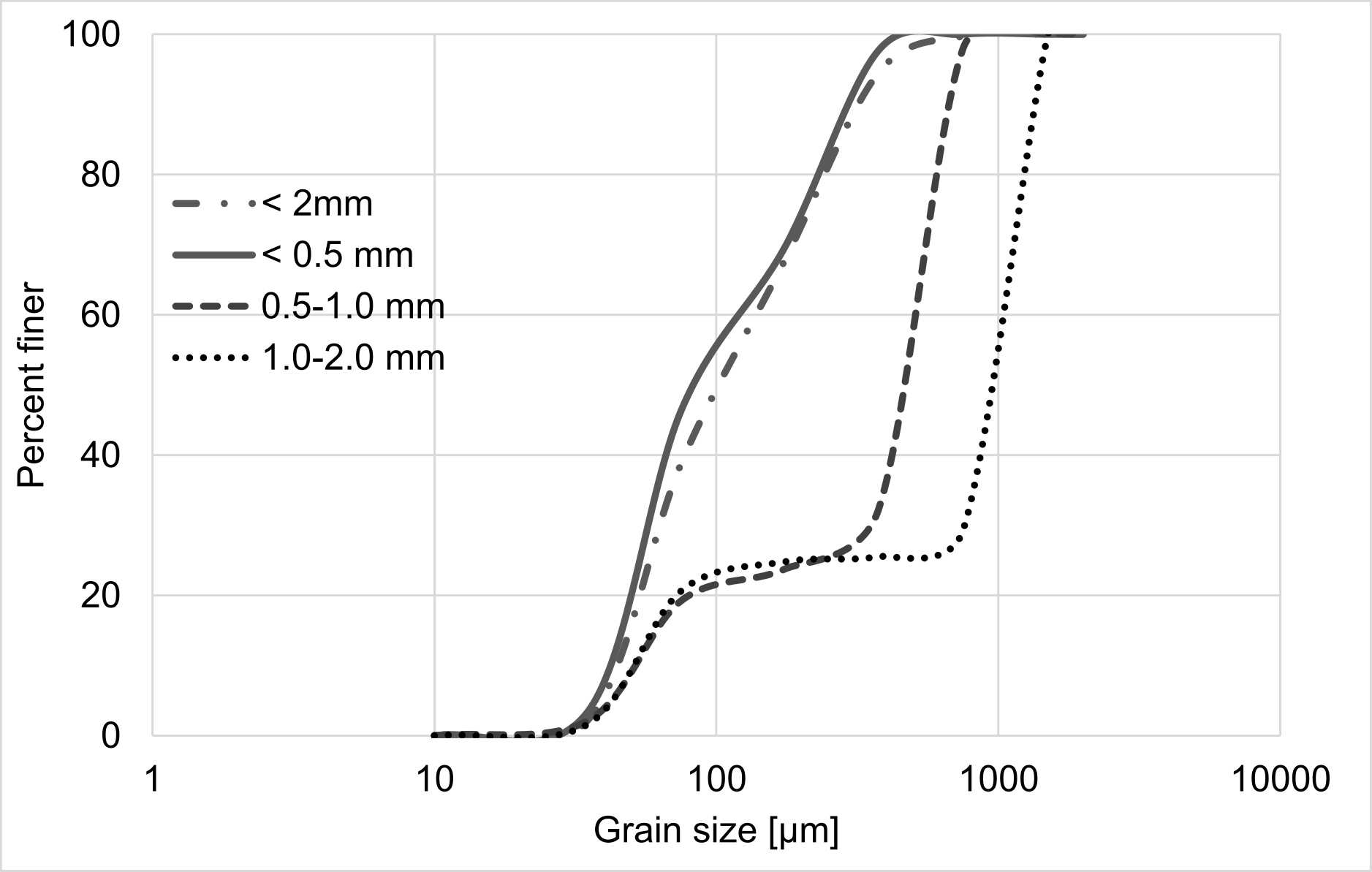

Supplement: S1 Fig — The dashed lines mean different sand fractions. (TIF) [file pone.0265546.s001.tif]

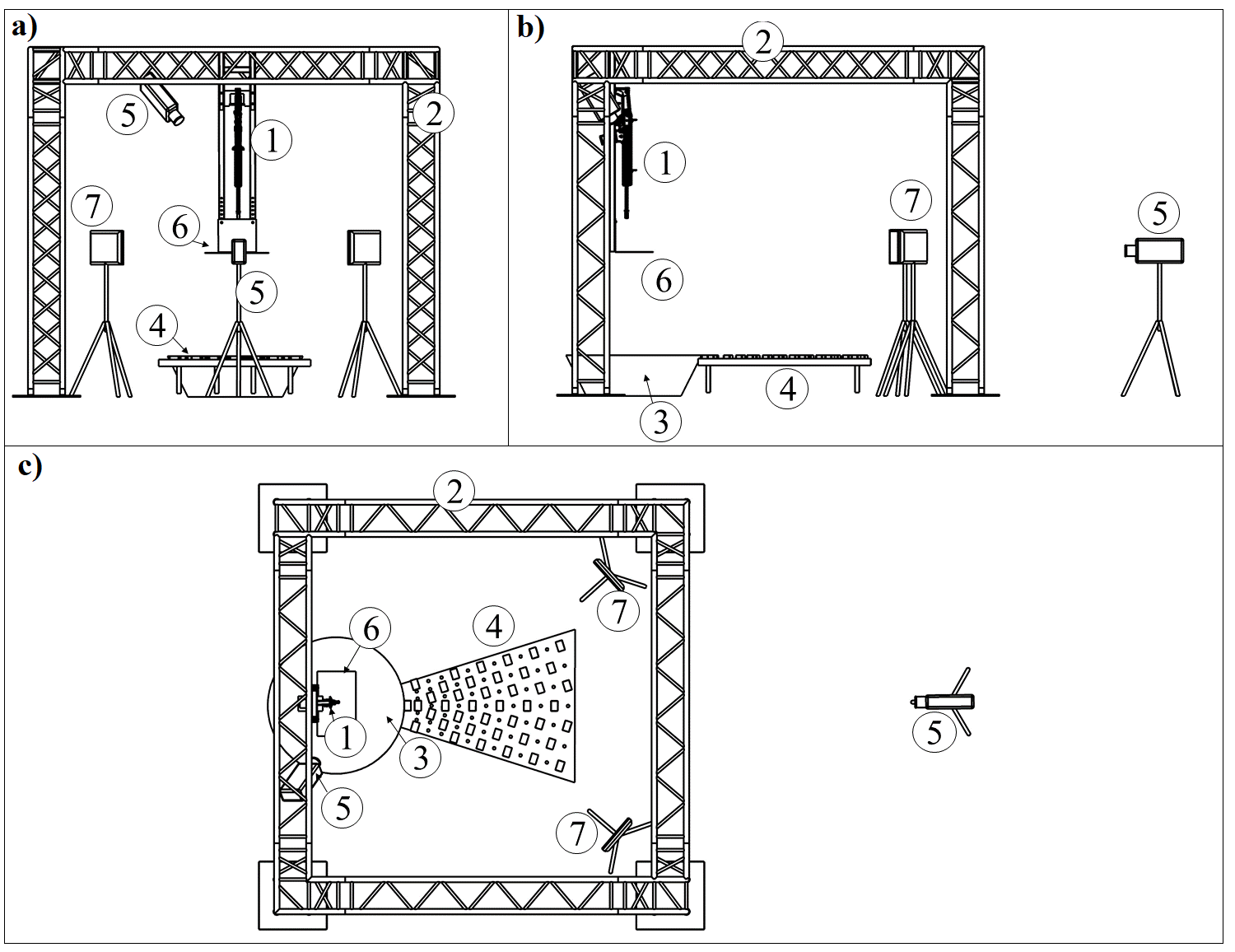

Supplement: S2 Fig — 1–rifle; 2–structure to ensure rifle stability during the shot; 3–sand container; 4–table with containers for collecting ejected grains of sand; 5–high-speed cameras; 6–protection plate against propellant gases; 7–front lighting LED lamp. (TIF) [file pone.0265546.s002.tif]
